# The role of Gut Microbiota in the development of obesity and Diabetes

**DOI:** 10.1186/s12944-016-0278-4

**Published:** 2016-06-18

**Authors:** Othman A. Baothman, Mazin A. Zamzami, Ibrahim Taher, Jehad Abubaker, Mohamed Abu-Farha

**Affiliations:** Department of Biochemistry, King Abdul Aziz University, Jeddah, Saudi Arabia; Faculty of Medicine, Aljouf University, Aljouf, Saudi Arabia; Biochemistry and Molecular Biology Unit, Dasman Diabetes Institute, Dasman, P.O. Box 1180, 15462 Kuwait City, Kuwait

## Abstract

Obesity and its associated complications like type 2 diabetes (T2D) are reaching epidemic stages. Increased food intake and lack of exercise are two main contributing factors. Recent work has been highlighting an increasingly more important role of gut microbiota in metabolic disorders. It’s well known that gut microbiota plays a major role in the development of food absorption and low grade inflammation, two key processes in obesity and diabetes. This review summarizes key discoveries during the past decade that established the role of gut microbiota in the development of obesity and diabetes. It will look at the role of key metabolites mainly the short chain fatty acids (SCFA) that are produced by gut microbiota and how they impact key metabolic pathways such as insulin signalling, incretin production as well as inflammation. It will further look at the possible ways to harness the beneficial aspects of the gut microbiota to combat these metabolic disorders and reduce their impact.

## Background

Obesity and its associated disorders have reached an alarming stage worldwide. The last decades have experienced an exponential increase in the number of people suffering from obesity and its associated disorders such as T2D [1–7]. Sedentary lifestyle and increased food consumption has been considered the main underlying causes for this obesity epidemic [8–10]. Environmental and genetic factors have also been implicated including changes in the gut microbiota to play a role in the development of metabolic disorders [11–17]. Gut microbiota describes all organisms living in the gastrointestinal (GI) tract. The majority of these organisms reside in the large intestine. These bacteria play important physiological role in vital processes such as digestion, vitamin synthesis and metabolism amongst others. Even though the exact mechanism linking gut microbiota to obesity is far from being very well understood, it’s well established that gut microbiota can increase energy production from diet, contribute to low-grade inflammation and regulate fatty acid tissue composition [11, 18, 19]. These processes as well as others have been proposed as the link between obesity and gut microbiota. However, the exact contribution of gut microbiota to the development of obesity and diabetes is not very clear due to many reasons including the complexity and diversity of gut microbes, ethnic variation in studied populations and large variations between individuals studied [14, 20]. Nonetheless, modulation of gut microbiota holds a tremendous therapeutic potential to treat the growing obesity epidemic especially when combined with diet and exercise [21–23]. This review shed some light on the recent work linking gut microbiota with obesity and diabetes and looks at possible ways to modulate gut microbiota to control the spread of obesity and diabetes.

## Origin and composition of gut micribiota

The human body contains trillions of microorganisms that inhabit our bodies during and after birth [24–26]. During the pregnancy, infant’s intestinal tract is free of microbes until exposed to maternal vaginal microbes during normal birth [27]. Infants born through Caesarian section are exposed to maternal skin bacteria altering their bacterial gut composition [27]. Feeding represents another source of microorganisms where breast fed babies have different gut microbiota composition than formula fed babies [27]. Introduction of solid food represents another shift in the composition of babies gut microbiota [28]. After that, gut microbiota remains relatively unchanged until old age where the composition changes again. Adult humans have more than 10 times the number of bacterial cells than the cells constituting the human body. Majority of microbiota in the GI tract are bacteria, nevertheless, viruses fungi and other microorganisms are still present [14]. Even though, individuals have unique microbiota composition, gut microbiota is mainly members of four phyla (*Firmicutes*, *Bacteroidetes*, *Actinobacteria* and *Proteobacteria*) [19]. As shown in Table 1, the large intestine contains the highest number of bacteria containing over 10^11^ bacteria per gram of intestinal content. The mouth contains 10^12^ followed by the Ileum containing 10^8^–10^9^ bacteria [29]. On the other hand, the jejunum harbors 10^5^–10^6^ while the stomach has the least number of bacteria 10^3^–10^4^ [29]. Even though we are still far from identifying, let alone characterizing all bacteria in our system, advancing molecular biology techniques such as next-generation sequencing has tremendously contributed to our understanding of the gut microbiota [30]. The use of gnotobiological methods to breed mice in a sterile environment provided an invaluable tool to understand the role of infecting controlled bacterial cultures and defined bacterial strains into animals. Studying their effect through various genomic and proteomic tools [29].

**Table 1:** Number of bacteria in different components of the gastrointestinal tract

Digestive Tract	Number of Bacteria
Mouth	10^12^
Stomach	10^3^–10^4^
Jejunum	10^5^–10^6^
Terminal Ileum	108–109
Large Intestine	10^11^ Per gram of intestinal contents

## Factors affecting gut microbiota composition

**Fig. 1:**
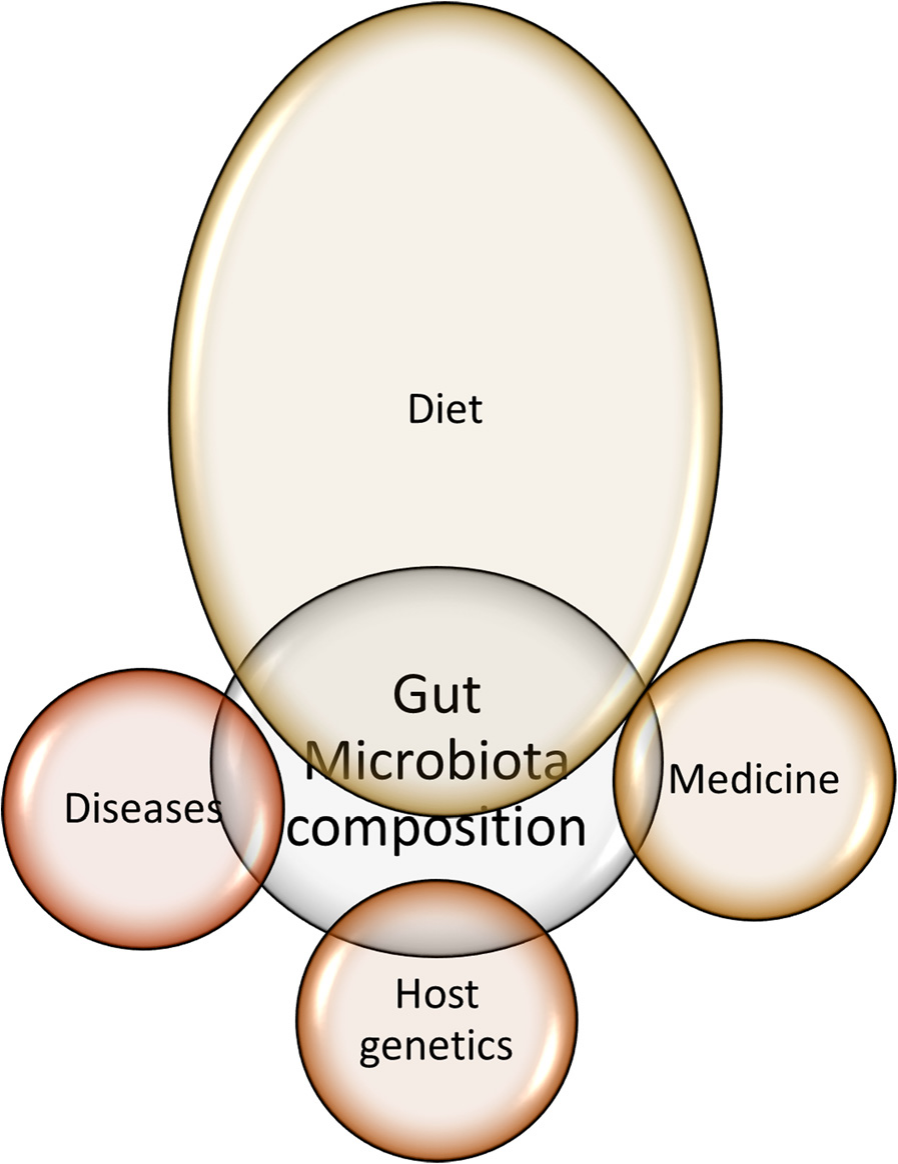
A diagram showing main factors affecting the gut microbiota composition highlighting the great impact of diet on this composition

Composition of gut microbiota is affected by many factors such as diet, disease state, medications as well as host genetics to name a few. As a result, the composition of the gut microbiota is constantly changing affecting the health and well-being of the host such as disease state as well as the use of various medicines such as antibiotics (Fig. 1). The effect of antibiotics on gut microbiota is well documented showing a long term reduction in bacterial diversity after use of antibiotics. Thuny et al has shown that the use of intravenous treatment by vancomycin plus gentamycin has been associated with a major and significant weight gain [31]. Link between antibiotics and weight gain is also well documented in infants as well, for example, Saari et al has linked antibiotic exposure during the first 6 months of age to weight gain in healthy children [32]. Furthermore, Studies have shown that the use of antibiotics will cause a decline in the bacterial diversity, stereotypic declines as well as increased abundances of certain taxa [33–43]. On the other hand, recovery of normal microbiota from certain antibiotic treatment can be long depending on the type of antibiotic and its spectrum [44]. Strong and broad spectrum antibiotics such as clindamycin can have longer affects persisting up to 4 years as suggested by some studies [45]. Moreover, the stress caused by the disruption of normal flora after antibiotic treatment facilitates the transfer of antibiotic resistance genes to virulent species leading to increased drug resistance [44]. These studies highlight the importance of better understanding of the role antibiotics play in modulating gut microbiota and their contribution to weight gain and potentially loss as well as other diseases.

Finally, the main contributor to the diversity of the gut microbiota is diet [46–52]. It has been suggested that changes in the diet can account for 57 % of the variations in microbiota compared to genetic variations in host that can only account for 12 % [53]. The effect of diet on microbiota composition is prominently observed as early as during breast and formula feeding as mentioned above. For example, level of *Bifidobacteria* spp. is higher in breast-fed babies compared to formula fed babies [54–59]. Formula-fed babies on the other hand have a more diverse microbiota with higher levels of *Bacteroids* spp. and *Lactobacillus* spp. [58]. Moreover, probiotics and prebiotics are among the most dietary strategies established for controlling the composition and metabolic activity of gut microbiota. Probiotics are non-pathogenic microorganisms used as food ingredients to benefit the hosts’ health. Jones et al investigated the effect of a bile salt-hydrolyase *Lactobacillus reuteri* strain in hypercholesterolemic individuals. They found this strain can significantly lower the low-density lipoprotein cholesterol (LDL-C) [60]. Also they proposed the role of nuclear receptor farnesoid X receptor (FXR) as transactional factor in reducing fat absorption from intestine. Furthermore, prebiotics are fermented dietary fibers have been shown to impact the host by specifically stimulating changes in the composition and/or activity of bacteria in the colon, and thus improving the hosts’ health [61]. Lactulose, resistant starch and inulin are the most prebiotic compounds used by the food industry to modify the composition of gut microbiota to benefit human health. These have been shown to mostly target bifidobacteria and lactobacilli [62, 63]. Prebiotics are carbohydrate-like compounds, such as lactulose and resistant starch, and have been used in the food industry to modify the composition of the microbiota species to benefit human health in recent years [62]. Inulin is one type of prebiotics. These prebiotics mostly target bifidobacteria and lactobacilli, which are two kinds of probiotics [63]. Recent research suggested that combining both prebiotics and probiotics, namely synbiotics can also fight obesity [64].

A number of studies have shown tight connection between diet and microbiota indicating how the composition of different diets will directly impact gut microbiota [47, 49, 51, 52]. In an earlier study, Turnbaugh et al used humanized mice that were generated by transplanting human feces into germ-free mice to study the effect of diet on microbiota [65]. Switching mice from low-fat, plant polysaccharide–rich diet to so call “Western diet”, a high-fat and sugar diet, altered the composition of the microbiota within a single day [65]. Mice fed with the Western diet had increased number of Erysipelotrichi class of bacteria within the Firmicutes phylum and reduced *Bacteroides* spp. Similarly mice fed a vegetarian diet, rich in dietary fibers, had lower counts of *Bacteroides* spp. *E. Coli* and other bacteria compared to the controls. Table 2 gives a summary of recent studies looking at changes in gut microbiota after consuming various types of diets that have various levels of sugar, fat and protein such as western diet, vegetarian and Calorie restricted diet.

**Table 2:** The effect of various diets on the composition of gut microbiota diversity

Diet Type	Effect on bacteria
High Fat Diet	Decrease of genera within the class Clostridia in the ileum. Increase Bacteroidales in large intestine [130]
	Increase Lactobacillus spp., Bifidobacterium spp., Bacteroides spp., and Enterococcus spp. Decrease Clostridium leptum and Enterobacter spp. [131]
	Increase Firmicutes to Bacteriodetes ratio. And increased Enterobecteriaceae [132]
	increase Bacteroidales, Clostridiales and Enterobacteriales [133]
Vegetarian Diet	Decrease Acteroides spp., Bifidobacterium spp., Escherichia coli and Enterobacteriaceae spp. [134]
	Decrease Enterobacteriaceae and increase Bacteroides [135]
	Increase Bacteroidetes, and decrease Firmicutes and Enterobacteriaceae [136]
Calorie restricted	Decrease Firmicutes to Bacteroidetes ratio [137]

## Obesity and gut microbiota

Due to the exponential increase in obesity rates and its associated complications such as diabetes in the past few decades, tremendous attention has been given to understanding underling mechanism. Albeit these tremendous efforts and the identification of candidate genes and mutations in studies like genome wide association studies (GWAS), full understanding is still lacking. During the last decade new studies have emerged suggesting a role for gut microbiota in the development of obesity and diabetes [11, 66–77]. More studies have been published showing a wide range role of gut microbiota in processes like energy homeostasis, blood circulation and autoimmunity to list a few. Early studies showed that obese mice as well as humans had different gut microbiota composition compared to lean. A number of studies showed an increase in bacteria from the Firmicutes phyla and a decrease in the Bacteroidetes phyla that is believed to be associated with increased energy absorption from food and increased low-grade inflammation [15, 17]. However, other studies showed no difference between these two phyla in lean and obese subjects, highlighting the need for focusing further on specific species within those groups rather than comparing them at the phyla level. Another example for the role of microbiota in obesity has been seen with patients undergoing Rouex-en-Y gastric bypass. After the surgery, patients observe dramatic metabolic improvement that cannot be explained by the caloric restriction and the weight loss alone. Changes in gut microbiota have been shown to play a role in this improvement as a shift in bacterial population has been observed in a number of studies [18–20, 76, 78–86]. In order to demonstrate the role of bariatric surgery in the changes of the gut microbiota, Liou et al showed that fecal transplantation from RYGB-treated mice into germ-free mice lead to weight loss and decreased fat mass in mice [87].

Gut microbiota contributes to energy metabolism through the production of SCFA that are produced by colonic fermentation which involves the anaerobic breakdown of dietary fiber, protein and peptides. SCFA are bacterial waste products that are produced by the bacteria to balance the redox state in the gut. Most abundant SCFA species are acetate, propionate, and butyrate. Acetate and propionate are mostly produced by Bacteroidetes phylum, while butyrate is produced by the Firmicutes phylum. They have been shown to exert beneficial effects on body weight, glucose homeostasis and insulin sensitivity. Gao et. al. showed that butyrate dietary supplementation reduces diet-induced insulin resistance in mice possibly through increasing energy expenditure and mitochondria function [88]. Butyrate and propionate were protective against diet-induced obesity [89]. Oral administration of acetate also improved glucose tolerance [90]. On the contrary to its proposed beneficial effect in diet induced obesity, cecal and fecal SCFA levels have been shown to be higher in genetically obese ob/ob mice and obese human subjects [16, 91, 92]. It has been suggested that this increase in SCFA is due to decreased colonic absorption with obesity [91].

SCFA can also act as signaling molecules and activate various pathways such as the activation of the AMP-activated protein kinase (AMPK) in liver and muscle tissues that triggers the activation of key factors involved in cholesterol, lipid, and glucose metabolism peroxisome proliferator-activated receptor-gamma coactivator 1 *alpha* (PGC-1α), Peroxisome proliferator-activated receptor gamma (PPARγ), and Liver X receptors (LXR) [93]. In addition SCFA have been also shown to activate Glucagon-like peptide-1 (GLP-1) through G-protein coupled receptor 43 (GPR43) which is also known as free fatty acid receptor 2 (FFAR2) [94, 95]. FFAR2 is one of the SCFA receptors and that has been shown to be activated by acetate and propionate followed by butyrate [96, 97]. Mice lacking the FFAR2 receptor were obese while its overexpression in adipose exhibited leanness under normal conditions [98]. It’s believed that these phenotypes were mediated by gut microbiota produced SCFA since these mice strains did not show the same phenotypes in mice grown under germ-free conditions or when treated with antibiotics [99]. The second SCFA receptor is GPR41, also called FFAR3 that shares 33 % amino acid sequence identity with FFAR2 and is activated mainly by propionate and butyrate [89]. Similar to FFAR2, FFAR3 is capable of inducing the gut hormone peptide YY (PYY) and GLP-1. It can also improve insulin signaling through SCFA produced by gut microbiota [100, 101].

Gut microbiota was also shown to play a role in the regulation of bile acids and cholesterol metabolism in both humans and animals [102]. Bile acids are synthesized in the liver by a multistep pathway. It can also act as an emulsifying agent in the intestine; helping to prepare dietary triacylglycerol and other complex lipids for degradation by pancreatic digestive enzymes. Before bile acids leave the liver, they convert into bile salts by conjugating to either glycine or taurine then re-absorbed in the ileum. A small amount of bile acids lost in fecal excretion via the action of intestinal bacteria. It was suggested that the possible role of gut microbiota in controlling bile acid and cholesterol metabolism might be induced by the up-regulation of transcription factors that link it to nutritional-induced inflammation, lipid absorption and *de novo* lipogenesis [102].

Low grade inflammation is a hallmark of obesity and T2D. Productions of pro-inflammatory cytokines are coordinated Via the Toll-like receptors (TLRs) and the master regulator of key inflammatory cascades the nuclear factor kappa (NF-kB) [103–106]. These pathways have been shown to be activated by the production of lipopolysaccharides (LPS) that are major component of the outer membrane of Gram-negative bacteria that is produced in the gut [106]. Higher LPS levels have been associated with increased fat intake. It was also observed in obese mice models. It has been proposed that dietary fat mediated the absorption of LPS linking them to obesity. In fact, it has been demonstrated that adding LPS to normal-diet induced insulin-resistance and lead to weight gain. It has been also shown that LPS binds to TLR4 receptor on macrophages and activate the production of inflammatory markers in a process that has been linked to impairing pancreatic β-cell by suppressing insulin secretion and decreasing gene expression of Pancreatic And Duodenal Homeobox 1 (PDX1) [107].

## Diabetes and gut microbiota

It’s becoming increasingly evident that gut microbiota is contributing to many human diseases including diabetes both type 1 and type 2. Type 1 diabetes (T1D) is an autoimmune disease that is caused by the destruction of pancreatic β-cells by the immune system. Even though T1D is mainly caused by genetic defect, epigenetic and environmental factors have been shown to play an important role in this disease. Higher rates of T1D incidence have been reported in recent years that are not explained by genetic factors and have been attributed to changes in our lifestyle such diet, hygiene, and antibiotic usage that can directly affect microbiota [108]. It has been shown that diabetes incidence in the germ free non-obese diabetic subjects or patients (NOD) was significantly increased which is in line with the observation that the rates of T1D is higher in countries with stringent hygiene practices [108]. Similarly comparison of the gut microbiota composition between children with high genetic risk for T1D and their age matched healthy controls showed less diverse and less dynamic microbiota in the risk group [109]. In the Diabetes Prevention and Prediction (DIPP) study it was shown that new-onset T1D subjects had different gut microbiota composition than controls [110]. They showed that in the control group, mucin synthesis was induced by lactate- and butyrate-producing bacteria to maintain gut integrity while mucin synthesis was prevented by the non-butyrate-producing lactate-utilizing bacteria leading to β-cell autoimmunity and T1D [110]. In another study linking intestinal microbes with the innate immune system Wen et al used Myd88 knockout to show that specific-pathogen free (SPF) NOD mice lacking MyD88 protein do not develop T1D [111]. MyD88 is a mediator for multiple innate immune receptors such as TLR4 that recognize microbial stimuli [112]. Many other studies confirmed the differences observed in gut microbiota composition between T1D and their matched health controls highlighting the need for better understanding of the role that these bacteria may play in the development of this disease [108, 109, 113–122].

The link between T2D and gut microbiota is becoming clearer with more studies showing the involvement of microbiota in obesity and their role in insulin signaling and low grade inflammation as discussed in the previous section. The effect of microbiota on T2D has been proposed to be mediated through mechanisms that involve modifications in the secretion butyrate and incretins [94, 95, 101, 123, 124]. Qin et al showed that T2D patients had moderate degree of gut microbial dysbiosis, a decrease in universal butyrate-producing bacteria and an increase in opportunistic pathogens [125]. Similar data were reported by other studies highlighting the role of these bacteria in regulating important T2D pathways such as insulin signaling, inflammation and glucose homeostasis [13, 18, 99, 124–129]. On the other hand, gut microbiota has been shown to affect the production of key insulin signaling molecules such as GLP-1 and PYY through SCFA and its binding to FFAR2 [123]. These two molecules have favorable effects, decreasing insulin resistance and the functionality of β-cells [123]. An increase in Bifidobacterium spp. in mice has been linked to have anti-inflammatory effect through the production of GLP2 and reducing intestinal permeability [124]. These are just a few examples on the potential impact of gut microbiota on the development of T2D.

## Conclusions

In conclusion, overwhelming evidence is available highlighting the important role of gut microbiota in key metabolic diseases impacting key pathways like energy homeostasis and inflammation. Changes in life style that involves increased food consumption and reduced exercise in addition to gut microbiota contribute more to metabolic diseases. As a result, better understanding and utilization of various prebiotic and probiotic bacteria may prove to be beneficial in the treatment of metabolic diseases in the future.
